# Development of an evidence-based framework to guide delegation of clinical tasks to physiotherapy support workers in musculoskeletal outpatient physiotherapy services

**DOI:** 10.1186/s12913-026-14210-0

**Published:** 2026-02-23

**Authors:** Panos Sarigiovannis, Nadine E. Foster, Sue Jowett, Benjamin Saunders

**Affiliations:** 1 School of Medicine, Keele University, Staffordshire, ST5 5BG UK; 2 Midlands Partnership University NHS Foundation Trust, St George’s Hospital, Stafford, Staffordshire, ST16 3SR UK; 3 STARS Education and Research Alliance, Surgical Treatment and Rehabilitation Service (STARS), The University of Queensland and Metro North Health, Herston, Brisbane, Australia; 4 Health Economics Unit, Institute of Applied Health Research, Public Health Building, University of Birmingham, Edgbaston, Birmingham, B15 2TT UK

**Keywords:** Delegation, Physiotherapy support workers, Musculoskeletal physiotherapy, Mixed methods, Triangulation, Healthcare workforce, Clinical framework

## Abstract

**Background:**

Delegation of clinical tasks to physiotherapy support workers (PSWs) is a key strategy in musculoskeletal (MSK) outpatient physiotherapy services to meet rising demand and optimise workforce use. However, delegation practices remain inconsistent, due to variability in training, role definition, supervision and patient communication. This paper presents the final phase of a mixed-methods research program. In this phase, findings from earlier phases were triangulated to inform the development of a practical, evidence-based framework to support safe and consistent delegation in MSK outpatient physiotherapy services.

**Methods:**

This final phase of the research program used a triangulation approach to integrate findings from three earlier phases: (1) a focused ethnography of real-world delegation practices; (2) a consensus study using nominal group technique to identify best practice components of a delegation framework; and (3) a discrete choice experiment capturing patient preferences. Triangulation followed Farmer et al.’s convergence coding matrix to assess agreement, partial agreement, silence, or dissonance across data sources. A component was included if supported by at least two phases.

**Results:**

Triangulation revealed strong convergence across professional and patient perspectives. Seven core components were identified for inclusion in the final framework: (1) training and development, (2) a clear delegation process, (3) defined roles, (4) a supportive team culture, (5) embedded safety mechanisms, (6) patient awareness and communication, and (7) implementation and evaluation strategies. The framework was developed with input from a clinical advisory group and public contributors to ensure relevance and applicability to real-world practice.

**Conclusions:**

This final phase of the research program synthesised diverse findings to produce a framework for improving delegation to PSWs in MSK physiotherapy services. The framework offers structured, practical guidance to support consistent delegation in clinical teams. The principles may be transferable to other healthcare settings. Further research should explore implementation and evaluate impact in routine practice.

## Introduction

Musculoskeletal (MSK) conditions, including low back pain and arthritis, are a leading cause of disability globally. In the United Kingdom (UK), they affect over 20 million people and are the second most common reason for sickness absence from work [1]. Most MSK care is delivered in primary and community settings, where physiotherapists play a key role in assessment and treatment planning [2]. Increasingly, physiotherapy support workers (PSWs) contribute to follow-up care, such as leading exercise sessions or providing one-to-one treatments.

The PSW role was introduced to help meet rising service demand, manage workforce shortages, and support the sustainability of the health workforce and service delivery. Eliassen et al. [3], Lizarondo [4], Munn [5]. PSWs are not professionally regulated, and their job titles, training, and responsibilities vary widely [6]. The [7] estimates that PSWs account for around 15% of the UK’s physiotherapy workforce, with the majority employed within the National Health Service (NHS). Despite their growing role, there remains a lack of clear national guidance on how to delegate clinical tasks to PSWs, leading to inconsistent practice, role ambiguity, and concerns about accountability, training, and patient communication [8, 9]. Inadequate delegation can result in confusion over responsibilities, missed care, delays in escalation, and erosion of patient trust, especially when patients are unaware of who is delivering their care. These issues can undermine both service efficiency and the quality of care, highlighting the need for an evidence-based framework to support safe and appropriate delegation.

To address these issues, the Musculoskeletal Outpatient Physiotherapy Delegation (MOPeD) program of research was established with the overall aim of developing an evidence-based delegation framework to guide physiotherapists, managers, and service leads in safe, effective, and patient-centred delegation. A framework can be defined as a conceptual structure that supports the development and application of practical or operational strategies [10]. The intention was to create a framework that promotes greater consistency in delegation practices across services while retaining flexibility for local adaptation, recognising that MSK physiotherapy outpatient services operate within diverse clinical, organisational, and geographic contexts. The research used an exploratory sequential mixed-methods design structured into four phases:

### Phase 1: Focused ethnography

This phase explored how delegation occurred in practice within two NHS Trusts. It involved over 150 h of non-participant observation, document analysis, and 30 semi-structured interviews with physiotherapists, physiotherapy managers, PSWs, and patients. The interviews were guided by a topic guide developed for the focused ethnography, which is detailed in two manuscripts: one published and one currently under review [11, 12]. The ethnography identified key contextual and cultural factors influencing delegation, such as role clarity, workplace trust, and the visibility of PSWs to patients.

### Phase 2a: Consensus study using the nominal group technique (NGT)

Building on findings from the ethnography, three professionally homogenous NGT workshops were conducted with physiotherapists (*n* = 7), PSWs (*n* = 10), and managers (*n* = 10). Each group identified and ranked essential components of effective delegation. This phase generated a prioritised list of best practice features, including training needs, role definitions, communication strategies, and governance processes.

### Phase 2b: Discrete choice experiment (DCE)

The DCE surveyed 382 patients from one NHS Trust to quantify their preferences for different configurations of outpatient physiotherapy care. Participants were presented with hypothetical service options that varied by provider type (physiotherapist vs. PSW), continuity of care, wait times, number of follow-ups, and proximity of service. The analysis showed that while most patients preferred physiotherapist-delivered care, patients who had previously been treated by PSWs were more likely to choose them again, provided other service features were acceptable or better than those involving physiotherapists. Further details about the research programme, as well as the findings from each phase are reported in separate publications [11–15]. This paper reports the final phase of the MOPeD research program, which involved the triangulation of findings from all three phases to develop a practical, evidence-informed framework for delegation in MSK outpatient services.

## Methods

### Triangulation approach

A triangulation approach was used to integrate findings from the three earlier phases of the MOPeD research program. In mixed methods research, triangulation refers to the integration of diverse methodological approaches to gain a more comprehensive understanding of a complex phenomenon [16, 17]. This process enhances the credibility of findings by comparing data across methods, sources, and perspectives. The triangulation process drew on the framework proposed by Farmer et al. [18], which involves identifying themes within each phase and comparing them using a convergence coding matrix. Each theme was assessed for the degree of alignment between the phases using four categories:

- Agreement: Themes aligned across studies in meaning and prominence.
- Partial agreement: Themes overlapped in meaning or emphasis, but not both.
- Silence: A theme was present in one phase but absent in another.
- Dissonance: Themes diverged significantly across phases.

This structured coding process enabled comparison of findings across qualitative and methods. It also supported integration across different participant groups, including patients, physiotherapists, support workers, and physiotherapy managers. The initial coding and classification were conducted by the lead author (PS). To enhance rigour, the coding framework was refined through discussions with all authors, who included experts in qualitative methods, physiotherapy, social sciences and health economics. Investigator triangulation was applied throughout, with multiple researchers reviewing and challenging interpretations during the process.

While the program of research was designed so that earlier phases informed subsequent ones, e.g., ethnographic insights guided attribute selection for the DCE, the triangulation phase involved a holistic synthesis of all findings of the three earlier phases. This final integrative phase enabled the identification of core components to inform the development of a best practice delegation framework. Ethical approval was granted by the South West - Frenchay NHS Research Ethics Committee and the UK Health Research Authority (REC reference 21/SW/0158, IRAS project 297095).

### Patient and public involvement and engagement (PPIE)

Seven individuals with lived experience of MSK physiotherapy and/or support worker care contributed to research phase design, ethical approval, and the review of participant-facing materials. Following data collection, they were actively involved in reviewing the triangulated themes and shaping the framework to reflect patient values and expectations.

### Clinical advisory group (CAG)

A group of practicing clinicians, including physiotherapists, physiotherapy managers and clinical leads, supported early research phase planning and ethical review. Following approval, the group continued to advise on interpretation and application of findings. During framework development, their role was to assess the feasibility and relevance of each proposed component within routine NHS MSK outpatient services.

### From themes to framework components

Following triangulation, themes that demonstrated agreement or partial agreement in at least two of the three phases were considered for inclusion in the delegation framework. Silence in one dataset did not preclude inclusion if supported by the others. Dissonant findings were not included. The decision-making process for translating themes into framework components was carried out collaboratively with the study’s Clinical Advisory Group and PPIE group. These stakeholders critically reviewed the triangulated themes and contributed to refining them into actionable and practically relevant components. Their input ensured that the resulting framework was grounded in real-world clinical and patient experience.

## Results

Seven themes emerged from all phases of the program. Table 1 summarises key findings from each phase, and Table 2 presents the triangulation assessment used to evaluate alignment between the phases.

**Table 1:** Convergence of findings

Theme	Focused ethnography findings	Consensus study findings	DCE findings
Clear Delegation Process	Highlighted the need for a clear and thorough delegation process as it facilitates delegation in clinical practice	Emphasised having a clear delegation process, clear reporting mechanisms, and structured handovers.	No data
Training and development	Identified the need for delegation training for both physiotherapists and support workers as crucial, especially for newly qualified staff and/or physiotherapy undergraduate students. Induction should include spending time observing how physiotherapists and physiotherapy support workers work.	Recommended training for both physiotherapy support workers and physiotherapists, protected time for CPD, regular appraisals, mentorship, and structured supervision for all staff.	No data
Role definition	Physiotherapists and physiotherapy managers in both services mentioned the importance of ensuring that support workers have a clear role and all staff are familiar with the different roles in the team	Called for clearly defined roles, scope, boundaries, and standardised job titles for support workers.	No data
Supportive cultural environment	Stressed the importance of a supportive culture fostering trust, respect, and open communication between physiotherapists and physiotherapy support workers.	Not mentioned directly but suggested structured support networks, equal CPD opportunities, and mentorship programs to enhance collaboration.	No data
Patient awareness and experience	Patients were unaware they were treated by support workers but were satisfied with the care provided. All patients treated by physiotherapy support workers were satisfied with their treatment	No data	Patients who were treated by physiotherapy support workers were more likely to accept them in future care indicating that they were satisfied with the care they received.
Safety nets	Physiotherapists and physiotherapy support workers highlighted the importance of having a safety net to refer patients back to the physiotherapist as a significant facilitator of delegation.	Recommended safety net to identify when patients need to be referred back to the physiotherapist and escalation processes for complex cases.	No data
Patient preferences	Patients prefer to be seen quickly when there is clinical need, preferable face to face, to have more follow ups, to go to clinics close to their home, with ample parking and to be seen by the same clinician	No data	Patients prefer to be seen quickly, to have one to one sessions, to have more follow ups, to go to clinics close to their home, with ample parking and to be seen by the same clinician. Patients treated by physiotherapy support workers are likely to choose to be treated by them again when other service characteristics are as good or better than in a service treated by physiotherapists.

**Table 2:** Triangulation assessment of themes emerging from the three phases^1^

Theme	Focused ethnography findings	Consensus study findings	DCE findings
Clear delegation process	Agreement	Agreement	Silence
Training and development	Agreement	Agreement	Silence
Role definition	Partial agreement	Partial agreement	Silence
Supportive cultural environment	Partial agreement	Partial agreement	Silence
Patient awareness and experience	Partial agreement	Silence	Partial agreement
Safety nets	Agreement	Agreement	Silence
Patient preferences	Partial agreement	Silence	Partial agreement

^1^Note: strongest convergence was observed in three themes (training and development, clear delegation process, and safety nets), with agreement across both the ethnography and consensus study. The remaining four themes showed partial agreement. All themes were silent in one phase

### Development of best practice delegation framework

No dissonance was identified across the different phases of the research program. Of the seven themes identified through triangulation, three demonstrated agreement across two of the phases, while the remaining four showed partial agreement. In all cases, each theme was silent in one of the three phases of the research program, meaning it was not identified in that phase. The seven triangulated themes were developed into the core components of the final delegation framework, following further input from the CAG and PPIE group. The resulting framework comprises:

The final delegation framework comprises seven core components:

- Training and development
- Clear delegation process
- Role definition
- Supportive cultural environment
- Safety nets
- Patient awareness and communication
- Implementation and evaluation

**Fig. 1:**
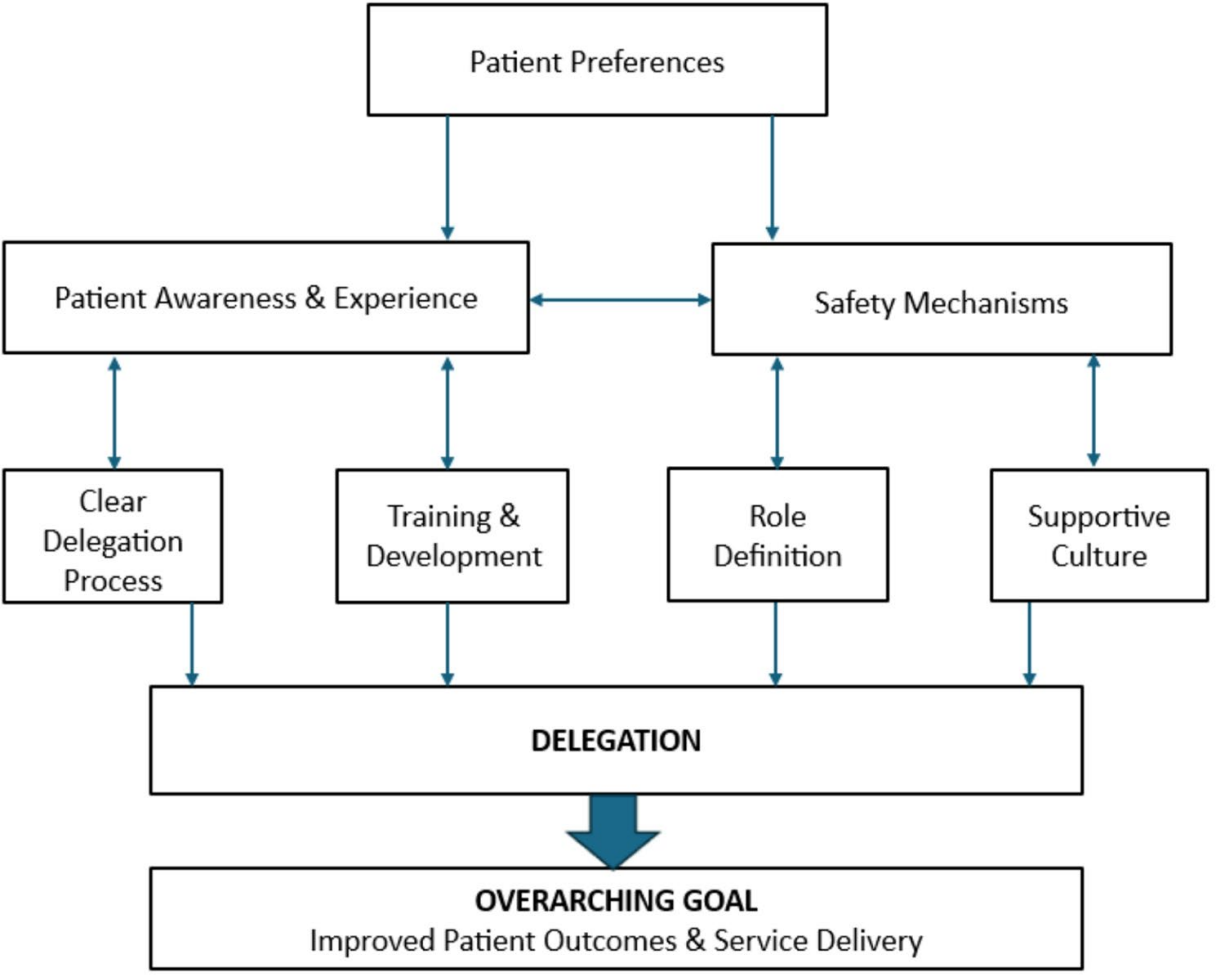
Development of a delegation framework to improve MSK physiotherapy service delivery and patient outcomes

**Fig. 2:**
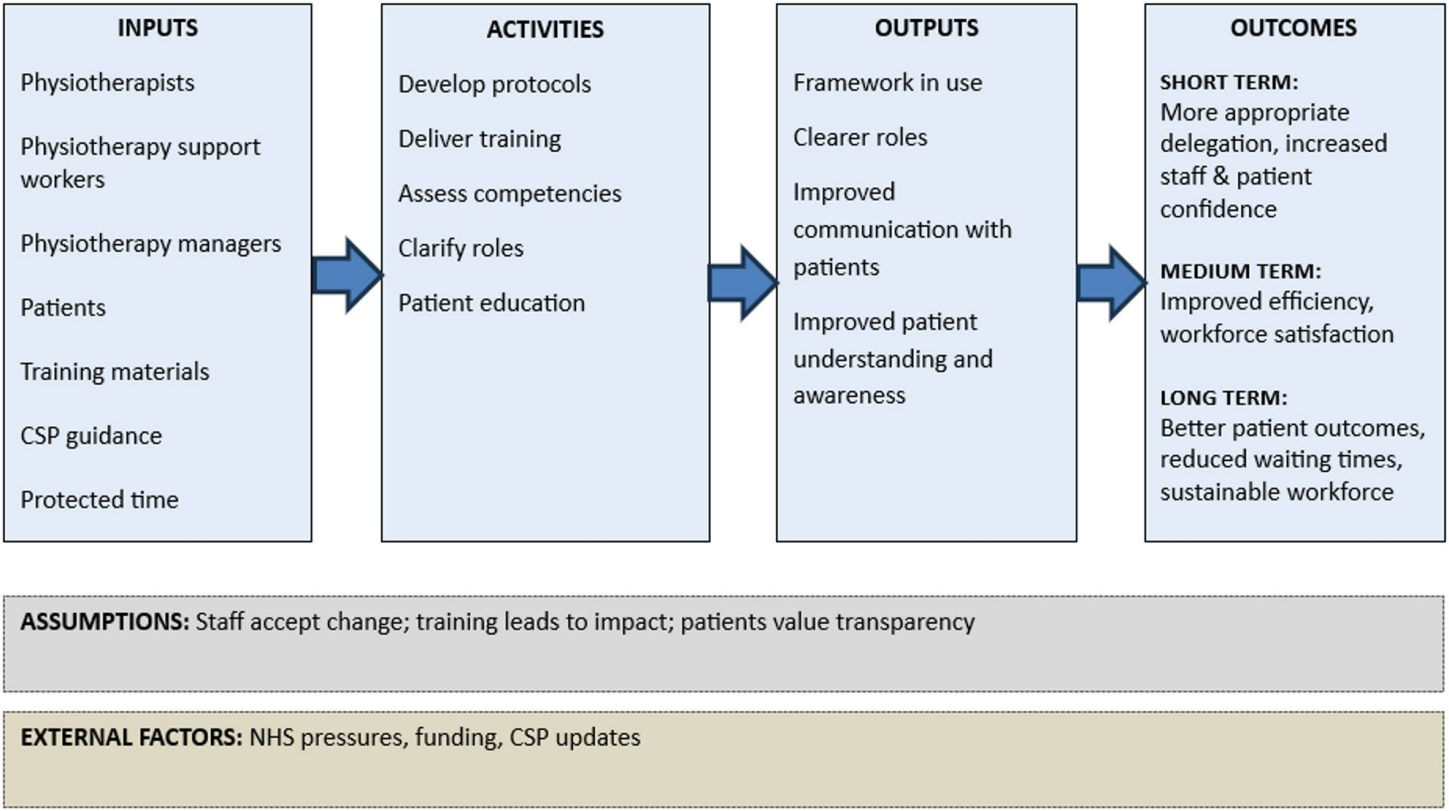
Logic model for delegation framework implementation

Table 3 presents the framework with supporting evidence from each phase and level of convergence across the three phases. While the framework is presented in tabular form, it is intended to be used as a practical tool by physiotherapy service leads, clinical managers, and physiotherapists to guide and implement evidence-informed delegation practices. For implementation, the framework is supported by two visual tools: a diagram showing its development (Fig. 1) and a logic model for local application and evaluation (Fig. 2). These resources are designed to support local adaptation and sustainable integration into clinical practice.

**Table 3:** The key elements of a best practice MSK physiotherapy delegation framework, with associated recommendations

Key elements	Recommendations
Training and development	• Offer specific training on delegation for newly qualified or new to the role clinicians and physiotherapy students • Competency-based training for physiotherapy support workers • Protected development time/CPD time for physiotherapists and physiotherapy support workers • Mentorship and supervision arrangements in relation to delegation of clinical tasks, particularly for physiotherapy support workers, newly qualified physiotherapists, physiotherapy students and physiotherapists new to the role • Induction of physiotherapists and physiotherapy support workers should include dedicated time for shadowing, allowing them to observe how physiotherapists and physiotherapy support workers operate in practice
Clear Delegation Process	• Competency-based task matching: Align tasks with the physiotherapy support worker’s training, experience, and demonstrated competencies • Provide physiotherapy support workers with clear instructions and expectations for delegated tasks • Mechanisms for clear reporting, handovers, and escalation • Thorough clinical documentation with regular audits
Role Definition	• Standardised job titles and role descriptions for physiotherapy support workers • Clear boundaries and scope of practice for physiotherapy support workers • Core skills and competencies defined and regularly updated for physiotherapists and physiotherapy support workers
Supportive Cultural Environment	• Fostering close working relationships based on trust, respect, and open communication • Structured support networks and equal CPD opportunities for all clinicians • Team-building initiatives to strengthen collaboration between physiotherapists and physiotherapy support workers
Safety Nets	• Mechanisms to identify appropriate patients for delegation • Clear escalation processes for complex cases or when patient needs exceed support workers’ scope • Standardised outcome measures to evaluate the effectiveness and safety of delegated care
Patient awareness and experience	• Ensuring physiotherapists and physiotherapy support workers clearly explain to patients when a physiotherapy support worker is treating them • Educating patients about support workers’ scope of practice, and role in care delivery • Mechanisms to gather and act upon patient feedback regarding patient experience with support workers
Patient preferences	• Consider continuity of care, and accessibility considerations such as clinic location and parking availability • Incorporating patient feedback to guide local delegation operating processes
Implementation and evaluation	• Implement the framework in selected MSK departments to assess feasibility and gather feedback • Evaluation Metrics: - Measure patient satisfaction, safety, and clinical outcomes - Assess physiotherapists’ and physiotherapy support workers’ performance, confidence and competency in delegation - Evaluate service efficiency, including reduced waiting times and optimised workflows

## Discussion

This final phase of the research program built on evidence from the three earlier phases to develop a framework for best practice in delegation within musculoskeletal outpatient physiotherapy. This phase employed triangulation to bring together qualitative and quantitative evidence to develop a best practice framework for the delegation of clinical tasks to PSWs in NHS MSK physiotherapy outpatient services. By systematically analysing findings from a focused ethnography, a consensus study, and a DCE, seven core components were considered essential for safe, effective, and consistent delegation. These included training and development, structured delegation processes, role clarity, a supportive team culture, safety mechanisms, patient communication, and implementation and evaluation strategies. Notably, strong agreement across the different phases of the research program reinforced the central importance of training for both physiotherapists and support workers, and of establishing clear protocols to guide delegation decisions.

The proposed framework presented here aligns with existing literature highlighting the importance of role clarity, structured communication, and targeted training and cultural factors such as trust, mutual respect, and open communication in effective delegation. Our findings reinforce prior research indicating the importance of role clarity, structured communication, and targeted training in effective delegation. Sarigiovannis et al. [8], in a review of delegation by Allied Health Professionals (AHPs) to support workers, highlighted the need for consistent training, clearer role definitions, and delegation support embedded in both undergraduate education and ongoing professional development. Etty et al. [19] similarly emphasised these requirements across the wider AHP workforce. Snowden et al. [20] identified that a lack of confidence and inconsistent delegation practices could be linked to inadequate preparation and variable supervision. Cultural factors, such as mutual respect, team trust, and effective interprofessional communication, were also identified as critical to successful delegation in earlier studies [21, 22]. The current framework builds on these findings by offering a structured, evidence-based approach tailored to MSK outpatient physiotherapy services. It also incorporates patient perspectives, which remain a relatively under-explored area in the delegation literature. The DCE results demonstrated that patients’ preferences are shaped not only by contextual features such as wait times, continuity, and convenience but also by who delivers care. This highlights the importance of transparent and responsive delegation practices.

Previous efforts to support delegation in healthcare have largely focused on broader workforce development. The Calderdale Framework Smith et al. [23], for example, is widely used across health and care settings to support task analysis and service redesign, including delegation. However, it is not profession-specific, and its emphasis on system-wide role review and competency mapping can make it less applicable for guiding everyday delegation decisions in outpatient clinical practice. In physiotherapy, early work by Saunders [24] highlighted important considerations for managing delegation, including the need for trust, clear communication, and appropriate supervision. While these foundational principles remain relevant, the context of modern physiotherapy has evolved substantially, with more diverse support worker roles, increased service demand, and shifting patient expectations. The Chartered Society of Physiotherapy has also published professional guidance on delegation and supervision [25, 26]. However, this guidance primarily addresses the competencies and limits of support worker roles and offers limited practical direction on how physiotherapists should approach delegation as a clinical decision-making process. As a result, delegation remains highly variable across services, shaped by individual confidence, local culture, and organisational norms [6].

### Implications for clinical practice and future research

This phase contributes new knowledge by developing an evidence-based framework that synthesises perspectives from clinicians, support workers, service leads, and patients. It builds on existing principles but responds directly to the lack of physiotherapy-specific tools that can support safe, effective, and consistent delegation in real-world outpatient settings. The triangulated findings have clear implications for clinical practice, workforce development, and professional guidance.

#### Establishing structured delegation processes

Services should implement clear delegation pathways that define roles, responsibilities, and scope of practice for both physiotherapists and PSWs. This can reduce variation, support accountability, and enhance team functioning.

#### Enhancing training and CPD

Delegation training should be embedded in physiotherapy undergraduate programs and continued through structured CPD for both PSWs and registered physiotherapists. PSWs require practical, role-specific education, while physiotherapists need training in supervisory and delegation skills [8, 19].

#### Updating professional guidance

Current guidance [25, 27] focuses primarily on PSWs’ competency but lacks emphasis on physiotherapists’ responsibilities as delegators. A dual-focus approach is needed to ensure both roles are supported with appropriate training, supervision and oversight mechanisms.

#### Fostering a supportive workplace culture

A culture of open communication, collaboration, and mutual respect supports safe and effective delegation. Structured mentorship, regular team meetings, and feedback mechanisms can promote shared learning and professional cohesion [27].

#### Promoting patient awareness and transparency

Clear communication with patients about who is treating them and the qualifications of their provider is vital for informed consent. Introducing PSWs during appointments, providing written information, and clarifying roles during consultations can enhance patient understanding and trust [28, 29].

#### Aligning delegation with patient preferences

Delegation models should consider patient priorities such as continuity of care, accessibility, and individualised treatment. These factors impact satisfaction and service uptake and should be integrated into workforce planning and service delivery [30, 31].

#### Embedding safety mechanisms

Clear escalation pathways are essential to ensure that delegated tasks remain within safe limits and that complex cases are promptly referred to registered physiotherapists. These safeguards are key to maintaining clinical quality and protecting patient wellbeing.

Successful implementation of the delegation framework may depend on the presence of enabling departmental structures. These include clear lines of accountability, defined roles, robust supervision systems, and a team culture that promotes openness and collaboration. Without such organisational foundations, the capacity to enact the framework’s recommendations may be limited.

Barriers to implementation may include resource constraints, such as time, staffing levels, and infrastructure, as well as the availability of physiotherapy support workers. A key precondition for success is ensuring sufficient PSW capacity, supported by appropriate induction, training, and career development opportunities. Evidence from our focused ethnography [12] highlighted this challenge. In one NHS service, long waiting lists for PSW appointments discouraged physiotherapists from delegating tasks, particularly when physiotherapists could offer follow-up appointments sooner than PSWs. This finding illustrates how insufficient PSW capacity can undermine the practical feasibility of delegation, even when clinical protocols are in place.

While some components of the framework may require upfront investment, there is potential for longer-term efficiency gains through improved use of skill mix and reduced service bottlenecks. Any future cost-effectiveness analyses should consider the system as a whole, including the potential to free up physiotherapists’ time to manage more complex cases.

Future research should focus on implementing and evaluating the delegation framework developed in this program. As a complex intervention, as defined by the updated Medical Research Council (MRC) and National Institute for Health Research (NIHR) guidance (Skivington et al. [32]), the proposed framework requires structured implementation strategies that are sensitive to local service contexts. Co-designing these strategies with clinicians, managers, and other stakeholders will help ensure the framework is acceptable, feasible, and adaptable across different settings. Once implemented, its impact on clinical efficiency, patient outcomes, and workforce utilisation should be assessed using robust methodologies. A hybrid effectiveness–implementation trial could be appropriate, allowing researchers to examine both practical integration and measurable outcomes in routine care.

Further work is also needed to strengthen patient understanding of delegated care. Although patients expressed willingness to be treated by PSWs when service features were favourable, awareness of these different types of professional roles remained limited. Developing and testing communication tools, such as structured patient information leaflets, digital resources, or guided discussions, could improve transparency and support informed consent. Longitudinal research is also recommended to explore how delegation affects patient experience and service delivery over time, and whether the framework proposed here requires adaptation in light of system-level changes or new models of care.

### Strengths and limitations

A key strength of this research lies in its triangulation design, which enabled the integration of findings from three previous research phases. This approach allowed for a multidimensional understanding of delegation practices in NHS MSK outpatient physiotherapy, combining professional, organisational, and patient perspectives. Triangulation enhanced the rigour by identifying convergence and divergence across datasets, improving the credibility and transferability of findings. The framework developed is grounded in diverse real-world contexts and reflects agreement or partial agreement across at least two of the three research phases. Involving both a CAG and public contributors throughout the process strengthened the relevance and practical applicability of the resulting framework. Importantly, this research addresses a recognised gap in physiotherapy by offering a structured yet flexible approach to support delegation decisions in routine practice.

Nonetheless, several limitations should be acknowledged. First, although the triangulation process enabled rich integration, some themes were silent in one or more datasets, which may reflect methodological differences rather than absence of relevance. Second, while the framework was designed with adaptability in mind, it has not yet been tested for feasibility or effectiveness in practice. Its success will depend on how well it can be implemented across diverse service settings. Future research should therefore explore acceptability, usability, and early outcomes, and consider iterative refinement based on implementation findings. Finally, the framework was developed within the specific context of NHS MSK physiotherapy services in England. Although its principles may be relevant to other settings, caution is needed in assuming generalisability without further testing.

## Conclusion

This study presents an evidence-based framework to guide the delegation of clinical tasks to PSWs in NHS musculoskeletal outpatient services. Developed from qualitative and quantitative insights across multiple stakeholder groups, the framework offers practical direction for safe and consistent delegation with implications for service delivery, workforce planning and professional education. Future work should focus on testing its feasibility and impact in different care settings and consider its broader application to other allied health and nursing professions where delegation supports team-based models of care.

## Data Availability

The datasets generated and/or analysed during the current phase of the research program are not publicly available in order to comply with the requirements of ethical approval, but are available from the corresponding author on reasonable request.

## Abbreviations

CAG: Clinical Advisory Group
CPD: Continuing Professional Development
DCE: Discrete Choice Experiment
MRC: Medical Research Council
MOPeD: Musculoskeletal Outpatient Physiotherapy Delegation
MSK: Musculoskeletal
NHS: National Health Service
NIHR: National Institute for Health and Care Research
PPIE: Patient and Public Involvement and Engagement
PSWs: Physiotherapy Support Workers
UK: United Kingdom
